# Risk factors associated with oral Human Papillomavirus (HPV) prevalence within a young adult population

**DOI:** 10.1186/s12889-024-18977-x

**Published:** 2024-06-03

**Authors:** Aimee F. Whitton, Gillian L. Knight, Elizabeth K. Marsh

**Affiliations:** 1 School of Science, University of Derby, Derby, DE22 1GB UK; 2 Division of Medical Sciences and Graduate Entry Medicine, School of Medicine, University of Nottingham, Royal Derby Hospital, Derby, DE22 3DT UK; 3 Academic Leadership and Student Experience, University of Wolverhampton, Wolverhampton, WV1 1LY UK

**Keywords:** Human papillomavirus, Risk factors, Prevalence, Viral load, Sexual behaviour

## Abstract

**Background:**

The prevalence of, and risk factors for, genital Human Papillomavirus (HPV) infections within the young adult population are well-established; the same is not known for oral HPV. This observational study aimed to determine oral HPV prevalence and abundance within a UK young adult population, and examine if sexual practices and established risk factors of oropharyngeal squamous cell carcinomas (OPSCCs) (such as smoking and alcohol consumption) influenced HPV prevalence.

**Methods:**

Convenience sampling was used to recruit a small sample of 452 UK-based young adults studying at a higher education (HE) institution to the study; the study was not powered. A highly sensitive real-time PCR HPV screening method was developed for the detection of multiple HPV subtypes from oral swabs. HPV-positive samples were subsequently screened by qPCR for viral subtypes HPV-6, HPV-11, HPV-16, HPV-18. Results were analysed by univariate and multivariate methods and stratified for gender, with lifestyle behaviour data collected via questionnaire. Socio-economic status was not captured within the questionnaire.

**Results:**

We found a high oral HPV prevalence of 22.79%, with a dominance of high-risk viral type HPV-16 (prevalence 19.12%; abundance average 1.08 × 10^5^ copies/million cells) detected within healthy young adults. Frequent smoking (*p* = .05), masturbation (*p* = .029), and engagement in multiple sexual activities (*p* = .057), were found to be associated with oral HPV prevalence, and HPV-16 prevalence, whilst behaviours traditionally associated with genital HPV were not.

**Conclusions:**

Our results strengthen the link between sexual practices and oral HPV transmission. We suggest that young adults should be considered high-risk for the contraction of oral HPV, although acknowledge that this sample of HE students may not be representative of the wider population. We show that high-risk HPV-16 is prevalent in the healthy population, as well as dominating within OPSCC; this study is one of the first to determine the dominance of oral HPV-16 prevalence and abundance within this population, presenting a clear need for greater awareness of oral HPV infections, and the risk factors for HPV-positive OPSCC within young adults.

**Supplementary Information:**

The online version contains supplementary material available at 10.1186/s12889-024-18977-x.

##  Background

The sexually transmitted virus Human Papillomavirus (HPV) notably causes cervical cancer and other cancers of the genital tract. However, for over 10 years, HPV infection has been associated with the worldwide trend of increasing incidence of a subset of oropharyngeal squamous cell carcinoma (OPSCC) in the tonsils and base of tongue, particularly in younger men [1]. Indeed, oral HPV infections are associated with a 50-fold increase in the risk of malignant transformation [2], and HPV-driven OPSCCs are dominated by the presence of high-risk viral type HPV-16 in 90% of these cancers [3].

Risk factors for classical, HPV-negative, OPSCC also appear to be risk factors for HPV-mediated OPSCC, including smoking and alcohol consumption [3]. Furthermore, sexual practices are a significant risk for HPV-positive OPSCC [4, 5], most likely by facilitating oral transmission of the virus. Indeed, it is proposed that a change of sexual behaviours towards oral sex may be driving the increasing numbers of younger patients presenting with the disease [1, 6]. Gender is the most significant risk factor for HPV-mediated OPSCC, with a significant proportion of the disease burden in men [6, 7], and men more likely to be infected with a high-risk virus [8]. Whilst this could be influenced by differences in smoking rates between genders, it is unlikely to reflect vaccination status; generally, individuals presenting with OPSCC are not yet part of the vaccinated cohort, and, although data are promising, it is not clear whether vaccination offers protection for HPV-mediated OPSCC [9]. It is not yet known if these differences represent a fundamental difference in the pathogenesis of viral infections between men and women.

Worldwide reports of the prevalence of oral HPV infections range from 5.5–7.7% [10, 11]. However, national studies have reported higher prevalence rates of up to 26% [7, 9, 12–15], suggesting a significant geographical divergence of prevalence. The reported rates seem to be highly dependent, and changeable, upon the screening method used for analysis, for which there is no established convention [16]. We therefore developed a highly sensitive real-time PCR method for assessing the presence of HPV in oral swabs, and utilised this to investigate the prevalence, viral type, and viral load of oral HPV, alongside self-reported lifestyle behaviours, within a young adult population in the UK. Here we report a high prevalence of oral HPV within the study sample, presenting a need for greater awareness of high-risk oral HPV infections, as well as associated lifestyle behaviours, and risk factors for OPSCC in this age group.

## Methods

### Ethical approval

This study was conducted in accordance with University of Derby research ethics policies and regulations. Ethical approval was given by the College of Life and Natural Sciences Research Ethics Committee at University of Derby (LSREC_1516_10). Full informed consent (print and signature) was given by all study participants.

### Data collection

A convenience sample of 452 student participants were recruited in two phases at University of Derby between 2016–2019, an enrolment rate of 76.7%. Inclusion criteria: healthy adults (≥ 18 years), residing in the East Midlands, UK, and enrolled on an undergraduate or postgraduate course at University of Derby. Participants were recruited from programmes across the College of Life and Natural Sciences (Sport Sciences, Biological Sciences, Psychology, Geography and Geology) and introduced to the study during lectures through a presentation by the research (not academic) team, and given an information sheet. No incentives were given for participation. Recruitment concluded upon time- and resource-saturation; the study was not powered. Enrolled participants provided full informed written consent, donated a pseudonymised (to facilitate study withdrawal) oral sample (a self-administered foam-tipped applicator to brush the inside of their cheeks for squamous epithelial cell collection), and privately completed a pseudonymised study-specific lifestyle questionnaire (unvalidated but informed by the Natsal questionnaire; supplementary information), designed to collect data on sample demographics, HPV vaccination status, and lifestyle behaviours associated with HPV-positive OPSCCs, such as smoking status, alcohol consumption, and sexual practice. 438 participants completed the questionnaire and provided an oral sample. The study did not require follow up.

### Laboratory procedures

Samples were washed in 2 mL of phosphate-buffered saline (PBS) and subjected to centrifugation at 350 × g for 10 m for cell pellet collection. DNeasy Blood & Tissue Kit [Qiagen] was used for DNA extraction, according to manufacturer’s instructions for cultured cells.

Samples were assessed for DNA viability with the house-keeping reference gene beta-actin. Reactions were composed of 2 × PrecisionFAST qPCR master mix [PrimerDesign Ltd], 300 nM each primer, 100 nM probe [custom made by PrimerDesign Ltd], with 5 µl pure DNA (various concentrations), and thermocycled by an ABI StepOne Plus (95 °C 2 m, followed by 40 cycles of 95 °C 5 s; 60 °C 20 s).

Viable samples were investigated for multiple HPV subtypes in the oral cavity using real-time PCR. MY09/11 consensus primers (forward-5’GCMCAGGGWCATAAYAATGG’3, reverse-5’CGTCCMARRGGAWACTGATC’3) detected the L1 region of the HPV genome [17]. Each reaction was composed of 2 × PrecisionFast SYBR green qPCR master mix [PrimerDesign Ltd], 200 nM each primer, and 8 ng DNA. Reactions were thermocycled by an ABI Step One Plus (95 °C 3 m, followed by 40 cycles of 95 °C 20 s; 53 °C 30 s; 72 °C 10 s).

HPV-positive samples were screened using type-specific real-time qPCR assays for HPV subtypes, HPV-6, HPV-11, HPV-16, HPV-18. Primer-probes were custom designed to detect the E6/E7 region of the HPV genome [PrimerDesign Ltd]. Reactions were composed of 2 × PrecisionFAST qPCR master mix [PrimerDesign Ltd], 300 nM each primer, 100 nM probe, with 5 µl pure DNA (various concentrations), and thermocycled by an ABI StepOne Plus (95 °C 2 m, followed by 50 cycles of 95 °C 5 s; 60 °C 20 s).

### Data analysis

HPV-positivity, viral type, and viral load were analysed alongside lifestyle risk behaviour data, and stratified for gender. Chi-square tests and Fisher’s Exact tests assessed categorical variables with two or more independent sub-levels, whilst Mann–Whitney U tests assessed non-parametric ordinal data in SPSS [IBM]. Benjamini–Hochberg correction (20% false discovery rate) was used within groups post hoc. Missing data values were excluded from analysis.

## Results

### Study sample

**Fig. 1:**
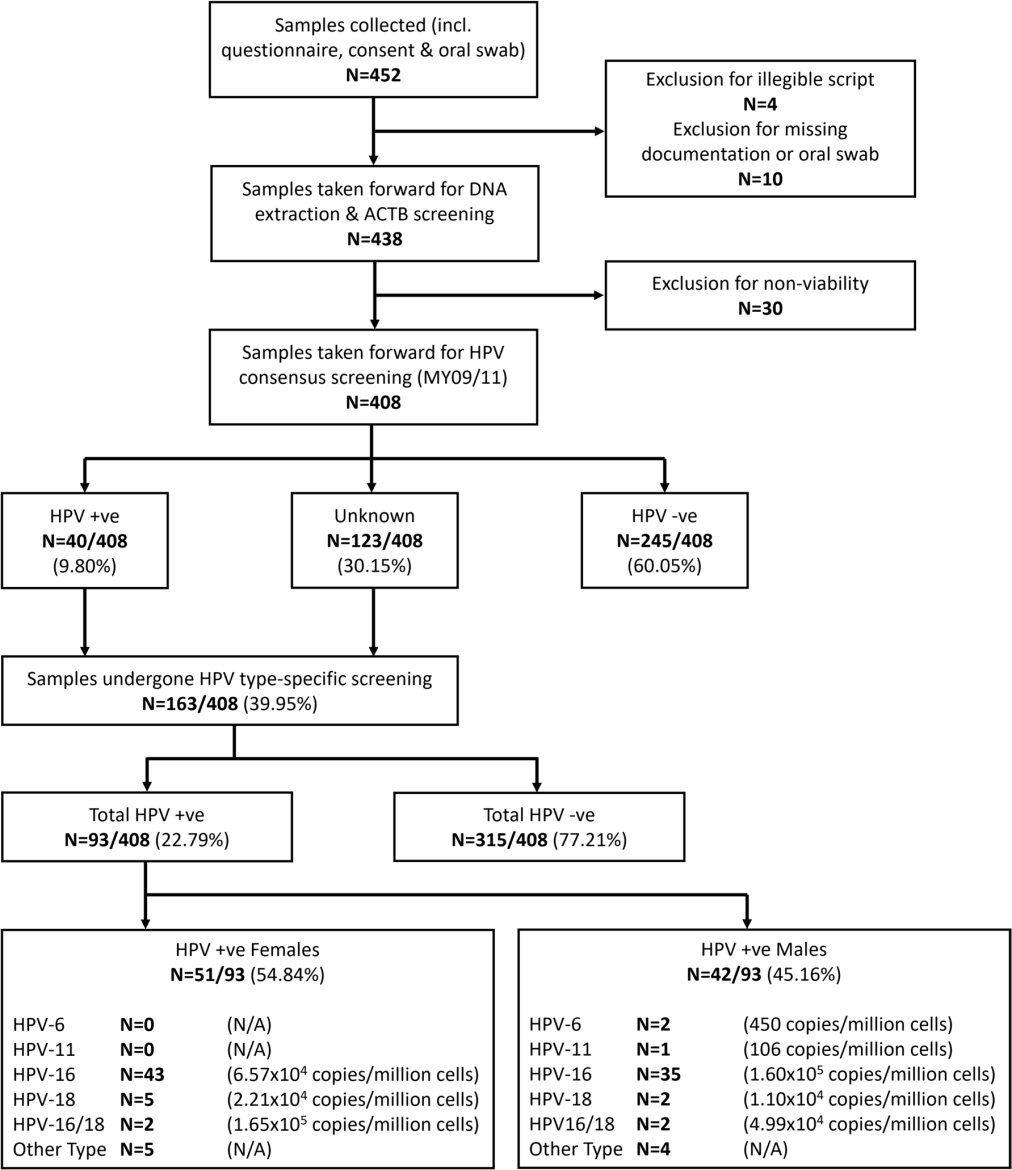
Sample selection and screening results flowchart. ACTB = Beta-actin housekeeping screening and real-time PCR absolute quantification for sample viability and cell number; HPV = Human papillomavirus consensus screening with MY09/11 primers and HPV-6, HPV-11, HPV-16 & HPV-18 type-specific probe-primers. Average calculated copy number/million cells per HPV type also shown

The study sample consisted of 452 participants but only 438 provided completed questionnaires to analyse (Fig. 1). Participants self-identified as either male (40.64%) or female (59.36%) rather than other offered identities, and 86.76% self-identified as ‘White’. 92.01% of the population were aged 18–25 years (Table 1).

**Table 1:** HPV screening results with demographics and lifestyle risk factors

	**HPV +ve** ***n* = 93**	**HPV –ve** ***n*= 315**	***p*-value**	**Adjusted*** p*-value^c^
**Demographics**				
Aged 18-25	85 (91.40)	291 (92.38)	.757	.2
Aged >25	8 (8.60)	24 (7.62)		
Ethnicity: White British	82 (88.17)	254 (80.63)	.094	.067
Ethnicity: Other White, BAME/BME & Unknown*	11 (11.83)	61 (19.37)		
Gender: Male	42 (45.16)	123 (39.05)	.291	.133
Gender: Female	51 (54.84)	192 (60.95)		
Gender: Other	0 (0.00)	0 (0.00)		
**Smoker Status**				
Current	17 (18.28)	61 (19.37)	.952	.2
Former	6 (6.45)	22 (6.98)		
Never	70 (75.27)	232 (73.65)		
**Smoking Frequency**	***n*= 23**	***n* = 78**		
Daily	15 (65.22)	32 (41.03)	.024b	**.05**
3-5 times/week	3 (13.04)	11 (14.03)		
1-2 times/week	3 (13.04)	6 (7.69)		
Few times/month	0 (0.00)	16 (20.51)		
Once a month	0 (0.00)	1 (1.28)		
Few times/year	2 (8.70)	12 (15.38)		
**Calculated Smoking Data**	***n*= 17**	***n* = 60**		
No. of cigarettes/day	6.88 ± 5.77 (0.01-20.00)	5.03 ± 5.30 (0.01-20.00)	.133b	**.15**
Pack Years	2.13 ± 2.82 (0.01-10.00)	1.75 ± 3.99 (0.01-27.00)	.079b	**.1**
**Alcohol Consumption Status**				
Current	87 (93.55)	274 (86.98)	.240a	.08
Former	2 (2.15)	11 (3.49)		
Never	4 (4.30)	30 (9.52)		
**Drinking Frequency**	***n*= 89**	***n* = 285**		
Daily	3 (3.37)	6 (2.11)	.454b	.16
3-5 times/week	9 (10.11)	28 (9.82)		
1-2 times/week	34 (38.20)	95 (33.33)		
Few times/month	19 (21.35)	83 (29.12)		
Once a month	10 (11.24)	20 (7.02)		
Few times/year	14 (15.73)	53 (18.60)		
**Types of Alcohol Consumed**	***n*= 89**	***n* = 285**		
≥2 Types of Alcohol Consumed	66 (74.16)	183 (64.21)	.082	.04
**Calculated Data**	***n*= 72**	***n* = 209**		
No. of units/week	16.02 ± 16.87 (1.00-71.00)	14.18 ± 15.44 (1.00-80.00)	.451b	.12
Binge Drinking	39 (54.17)	111 (53.11)	.877	.2
**Relationship Status**				
Single	41 (44.09)	133 (42.22)	.925	.2
Short-term (<1 year)	14 (15.05)	52 (16.51)		
Long-term/Married (≥1 year)	38 (40.86)	130 (41.27)		
**Sexual Orientation**				
Heterosexual	78 (83.87)	259 (82.22)	.531a	.114
Homosexual	5 (5.38)	9 (2.86)		
Bisexual	6 (6.45)	29 (9.21)		
Other/Unknown	4 (4.30)	18 (5.71)		
**Sexual Practice Descriptors**				
Open-Mouth Kissing	86 (92.47)	269 (85.40)	.075	**.086**
Ever had Sexual Intercourse	82 (88.17)	267 (84.76)	.411	.057
Within the last year†	76 (92.68)	245 (91.76)	.788	.143
STI status†	7 (8.54)	17 (6.37)	.497	.086
**Sexual Partners**	***n*= 82**	***n* = 266**		
1-5	54 (65.85)	187 (70.30)	.862	.171
6-10	15 (18.29)	45 (16.92)		
11-20	7 (8.54)	17 (6.39)		
>20	6 (7.32)	17 (6.39)		
**Sexual Activity**	***n*= 82**	***n* = 265**		
Vaginal Sex	79 (96.34)	259 (97.74)	.446a	.2
Anal Sex	26 (31.71)	69 (26.04)	.314	.143
Oral Sex	77 (93.90)	234 (88.30)	.146	.114
Foreplay	75 (91.46)	232 (87.55)	.332	.171
Masturbation	65 (79.27)	176 (66.42)	.027	**.029**
**Total Sexual Activities Engaged In**	***n*= 82**	***n* = 265**		
One	1 (1.22)	14 (5.28)	.043b	**.057**
Two	5 (6.10)	18 (6.79)		
Three	15 (18.29)	62 (23.40)		
Four	39 (47.56)	121 (45.66)		
Five	22 (26.83)	50 (18.87)		
**Condom Use**	***n* = 78**	***n* = 250**		
Never	30 (38.46)	75 (30.00)	.334b	.023
Sometimes (~25%)	22 (28.21)	85 (34.00)		
Mostly (~75%)	12 (15.38)	44 (17.60)		
Always	14 (17.95)	46 (18.40)		
**HPV Vaccination Status**				
Yes	34 (36.56)	133 (42.22)	.589	.2
No	50 (53.76)	151 (47.94)		
Unsure	9 (9.68)	31 (9.84)		

Data shown via count and percentage within HPV status group (%), or mean ± standard deviation (range). All data analysed using Chi-square tests for categorical observations, unless indicated. Denominators vary across variables because of item non-response

*HPV* Human Papillomavirus, *STI* sexually transmitted infection

^a^Fisher’s Exact test used due to expected counts being < 5

^b^Mann Whitney U test used for non-parametric continuous or ordinal ranked data

^c^After Benjamini–Hochberg post-hoc ranking; significance denoted in bold

*Grouped for statistical analysis due to large proportion of self-identified White British participants

†Data shown for sexually active group; *n* = 369 (+ ve = 82; –ve = 267)

### Oral HPV prevalence and abundance

Four hundred fifty-two oral swab samples were screened for viability using beta-actin, with 408 participant samples subsequently taken forward for HPV screening to examine viral prevalence, type, and abundance (Fig. 1). An overall prevalence of 22.79% (*n* = 93/408) was determined, with 0.49% (*n* = 2/408) prevalence attributed to HPV-6; 0.25% (*n* = 1/408) attributed to HPV-11; 19.12% (*n* = 78/408) attributed to HPV-16; and 1.72% (*n* = 7/408) attributed to HPV-18. Therefore, high-risk HPV-16 was the most prevalent (83.87%; *n* = 78/93) and abundant (average 1.08 × 10^5^ copies/million cells [range 5.12 × 10^3^–2.10 × 10^6^ copies/million cells]) viral type in the HPV-positive group. The second most prevalent (7.53%; *n* = 7/93) and abundant (average 1.89 × 10^4^ copies/million cells [range 1.27 × 10^2^–5.13 × 10^4^ copies/million cells]) viral type was high-risk HPV-18.

There was no significant difference in oral HPV prevalence between HPV-positive men and women (*p* = 0.291), or abundance (M = 1.48 × 10^5^ copies/million cells; *F* = 6.38 × 10^4^ copies/million cells, *p* = 0.602); but high-risk HPV types (HPV-16 and HPV-18) were detected in both groups, whereas HPV-6 and HPV-11 were only detected in men (Fig. 1).

### Risk factors for oral HPV

Age and ethnicity were not associated with oral HPV prevalence (Table 1). There was also no significant difference in oral HPV prevalence between the smoking and non-smoking populations, but frequent smokers had higher oral prevalence (*p* = 0.05), with the number of cigarettes smoked per day, and pack years playing a particular role (Table 1). Frequent smoking was also significantly associated with oral HPV-16 (*p* = 0.05) (Supplementary Table 1). Gender itself was not a risk factor; risk factors associated with gender are explored separately (Tables 2 and 3).

**Table 2:** Female HPV screening results and lifestyle behaviours

	**HPV +ve ** ***n* = 51**	**HPV –ve** ***n*= 192**	***p*-value**	Adjusted *p*-value^c^
**Smoker Status**				
Current	9 (17.65)	33 (17.19)	1a	.2
Former	4 (7.84)	15 (7.81)		
Never	38 (74.51)	144 (75.00)		
**Smoking Frequency**	***n*= 13**	***n* = 47**		
Daily	9 (69.23)	18 (38.30)	.016b	**.05**
3-5 times/week	2 (15.38)	8 (17.02)		
1-2 times/week	2 (15.38)	2 (4.26)		
Few times/month	0 (0.00)	0 (0.00)		
Once a month	0 (0.00)	9 (19.15)		
Few times/year	0 (0.00)	10 (21.28)		
**Calculated Smoking Data**	***n*= 13**	***n* = 42**		
No. of cigarettes/day	7.95 ± 5.92 (0.01-20.00)	4.81 ± 5.09 (0.01-20.00)	.053b	**.1**
Pack Years*	2.98 ± 3.43 (0.01-10.00)	2.03 ± 5.08 (0.01-27.00)	.058b	**.15**
**Alcohol Consumption Status**				
Current	50 (98.04)	164 (85.42)	.017a	**.04**
Former	1 (1.96)	8 (4.17)		
Never	0 (0.00)	20 (10.42)		
**Drinking Frequency**	***n*= 51**	***n* = 172**		
Daily	2 (3.92)	2 (1.16)	.958b	.16
3-5 times/week	1 (1.96)	10 (5.81)		
1-2 times/week	19 (37.25)	60 (34.88)		
Few times/month	10 (19.61)	46 (26.74)		
Once a month	10 (19.61)	13 (7.56)		
Few times/year	9 (17.65)	41 (23.84)		
**Types of Alcohol Consumed**	***n*= 51**	***n* = 172**		
≥2 Types of Alcohol Consumed	35 (68.63)	112 (65.12)	.642	.08
**Calculated Data**	***n*= 42**	***n* = 115**		
No. of units/week	12.49 ± 14.79 (1.00-71.00)	12.11 ± 14.43 (1.00-74.00)	.965b	.2
Binge Drinking	21 (50.00)	54 (46.96)	.735	.12
**Relationship Status**				
Single	21 (41.18)	80 (41.67)	.573	.143
Short-term (<1 year)	5 (9.80)	29 (15.10)		
Long-term/Married (≥1 year)	25 (49.02)	83 (43.23)		
**Sexual Orientation**				
Heterosexual	40 (78.43)	150 (78.13)	.657a	.171
Homosexual	3 (5.88)	5 (2.60)		
Bisexual	5 (9.80)	24 (12.50)		
Other/Unknown	3 (5.88)	13 (6.77)		
**Sexual Practice Descriptors**				
Open-Mouth Kissing	49 (96.08)	159 (82.81)	.016	**.029**
Ever had Sexual Intercourse	46 (90.20)	160 (83.33)	.225	.086
Within the last year†	45 (97.83)	144 (90.00)	.128a	.057
STI status†	3 (6.52)	10 (6.25)	1a	.2
**Sexual Partners**	***n*= 46**	***n* = 159**		
1-5	34 (73.91)	115 (72.33)	.536a	.114
6-10	5 (10.87)	28 (17.61)		
11-20	5 (10.87)	10 (6.29)		
>20	2 (4.35)	6 (3.77)		
**Sexual Activity**	***n*= 46**	***n* = 158**		
Vaginal Sex	46 (100.00)	158 (100.00)	d	
Anal Sex	10 (21.74)	32 (20.25)	.826	.171
Oral Sex	43 (93.48)	136 (86.08)	.178	.114
Foreplay	42 (91.30)	135 (85.44)	.302	.143
Masturbation	29 (63.04)	80 (50.63)	.138	.086
**Total Sexual Activities Engaged In**	***n*= 46**	***n* = 158**		
One	1 (2.17)	10 (6.33)	.100b	.057
Two	5 (10.87)	14 (8.86)		
Three	10 (21.74)	53 (33.54)		
Four	21 (45.65)	61 (38.61)		
Five	9 (19.57)	20 (12.66)		
**Condom Use**	***n* = 45**	***n* = 149**		
Never	21 (46.67)	47 (31.54)	.107b	.023
Sometimes (~25%)	12 (26.67)	46 (30.87)		
Mostly (~75%)	4 (8.89)	28 (18.79)		
Always	8 (17.78)	28 (18.79)		
**HPV Vaccination Status**				
Yes	34 (66.67)	132 (68.75)	.819	.2
No	12 (23.53)	38 (19.79)		
Unsure	5 (9.80)	22 (11.46)		

Data shown via count and percentage within HPV status group (%), or mean ± standard deviation (range). All data analysed using Chi-square tests for categorical observations, unless indicated. Denominators vary across variables because of item non-response

*HPV* Human Papillomavirus, *STI* sexually transmitted infection

^a^Fisher’s Exact test used due to expected counts of < 5

^b^Mann Whitney U test used for non-parametric continuous or ordinal ranked data

^c^After Benjamini–Hochberg post-hoc ranking; significance denoted in bold

^d^Statistical analysis not possible

*Calculated for smokers that provided information on number of years spent smoking; *n* = 41 (+ ve = 10; –ve = 31)

†Data shown for sexually active group; *n* = 206 (+ ve = 46; –ve = 160)

**Table 3:** Male HPV screening results and lifestyle behaviours

	**HPV +ve ** ***n* = 42**	**HPV –ve** ***n*= 123**	***p*-value**	Adjusted *p*-value^c^
**Smoker Status**				
Current	8 (19.05)	28 (22.76)	.899a	.075
Former	2 (4.76)	7 (5.69)		
Never	32 (76.19)	88 (71.54)		
**Smoking Frequency**	***n*= 10**	***n* = 31**		
Daily	6 (60.00)	14 (45.16)	.571b	.05
3-5 times/week	1 (10.00)	3 (9.68)		
1-2 times/week	1 (10.00)	4 (12.90)		
Few times/month	0 (0.00)	7 (22.58)		
Once a month	0 (0.00)	1 (3.23)		
Few times/year	2 (20.00)	2 (6.45)		
**Calculated Smoking Data**	***n*= 10**	***n* = 32**		
No. of cigarettes/day	5.48 ± 5.57 (0.01-17.00)	5.31 ± 5.63 (0.01-20.00)	.948b	.1
Pack Years*	0.92 ± 0.84 (0.01-2.00)	1.45 ± 2.40 (0.01-9.00)	.718b	.05
**Alcohol Consumption Status**				
Current	37 (88.10)	110 (89.43)	.895a	.16
Former	1 (2.38)	3 (2.44)		
Never	4 (9.52)	10 (8.13)		
**Drinking Frequency**	***n*= 38**	***n* = 113**		
Daily	1 (2.63)	4 (3.54)	.300b	.12
3-5 times/week	8 (21.05)	18 (15.93)		
1-2 times/week	15 (39.47)	35 (30.97)		
Few times/month	9 (23.68)	37 (32.74)		
Once a month	0 (0.00)	7 (6.19)		
Few times/year	5 (13.16)	12 (10.62)		
**Types of Alcohol Consumed**	***n*= 38**	***n* = 113**		
≥2 Types of Alcohol Consumed	31 (81.58)	71 (62.83)	.033	**.04**
**Calculated Data**	***n*= 30**	***n* = 94**		
No. of units/week	20.95 ± 18.56 (1.00-70.00)	16.72 ± 16.31 (1.00-80.00)	.190b	.08
Binge Drinking	18 (60.00)	57 (60.64)	.950	.02
**Relationship Status**				
Single	20 (47.62)	53 (43.09)	.698	.143
Short-term (<1 year)	9 (21.43)	23 (18.70)		
Long-term/Married (≥1 year)	13 (30.95)	47 (38.21)		
**Sexual Orientation**				
Heterosexual	38 (90.48)	109 (88.62)	.947a	.2
Homosexual	2 (4.76)	4 (3.25)		
Bisexual	1 (2.38)	5 (4.07)		
Other/Unknown	1 (2.38)	5 (4.07)		
**Sexual Practice Descriptors**				
Open-Mouth Kissing	37 (88.10)	110 (89.43)	.780a	.171
Ever had Sexual Intercourse	36 (85.71)	107 (86.99)	.833	.171
Within the last year†	31 (86.11)	101 (94.39)	.145a	.023
STI status†	4 (11.11)	7 (6.54)	.469a	.086
**Sexual Partners**	***n*= 36**	***n* = 107**		
1-5	20 (55.56)	72 (67.29)	.428a	.057
6-10	10 (27.78)	17 (15.89)		
11-20	2 (5.56)	7 (6.54)		
>20	4 (11.11)	11 (10.28)		
**Sexual Activity**	***n*= 36**	***n* = 107**		
Vaginal Sex	33 (91.67)	101 (94.39)	.692a	.114
Anal Sex	16 (44.44)	37 (34.58)	.289	.057
Oral Sex	34 (94.44)	98 (91.59)	.730a	.143
Foreplay	33 (91.67)	97 (90.65)	1a	.2
Masturbation	36 (100.00)	96 (89.72)	.066a	.029
**Total Sexual Activities Engaged In**	***n*= 36**	***n* = 107**		
One	0 (0.00)	4 (3.74)	.361b	.086
Two	0 (0.00)	4 (3.74)		
Three	5 (13.89)	9 (8.41)		
Four	18 (50.00)	60 (56.07)		
Five	13 (36.11)	30 (28.04)		
**Condom Use**	***n* = 33**	***n* = 101**		
Never	9 (27.27)	28 (27.72)	.645b	.114
Sometimes (~25%)	10 (30.30)	39 (38.61)		
Mostly (~75%)	8 (24.24)	16 (15.84)		
Always	6 (18.18)	18 (17.82)		
**HPV Vaccination Status**				
Yes	0 (0.00)	1 (0.81)	d	
No	38 (90.48)	113 (91.87)		
Unsure	4 (9.52)	9 (7.32)		

Data shown via count and percentage within HPV status group (%), or mean ± standard deviation (range). All data analysed using Chi-square tests for categorical observations, unless indicated. Denominators vary across variables because of item non-response

*HPV* Human Papillomavirus, *STI* sexually transmitted infection

^a^Fisher’s Exact test used due to expected counts of < 5

^b^Mann Whitney U test used for non-parametric continuous or ordinal ranked data

^c^After Benjamini–Hochberg post-hoc ranking; significance denoted in bold

^d^Statistical analysis not possible

*Calculated for smokers that provided information on number of years spent smoking; *n* = 36 (+ ve = 7; –ve = 29)

†Data shown for sexually active group; *n* = 143 (+ ve = 36; –ve = 107)

Alcohol consumption (status, unit intake, and frequency), relationship status, and self-identified sexual orientation were not associated with oral HPV prevalence of any viral type (Table 1). When examining sexual practice, a larger proportion of HPV-positive participants engaged in open-mouth kissing (*p* = 0.086), and oral sex (*p* = 0.114), but the association of these behaviours did not reach significance.

Lifestyle behaviours associated with genital HPV infection such as increased numbers of sexual partners, condom usage, and STI status were also not associated with oral HPV; but masturbation (*p* = 0.029), and an increasing diversity of sexual acts engaged with (*p* = 0.057) were associated with oral HPV prevalence (Table 1), and prevalence of HPV-16 alone (Supplementary Table 1). In the HPV-positive sample, 90.24% and 31.71% engaged in oral-vaginal and oral-anal activity, respectively, equating to 28.05% engaging in oral-genital activity overall. HPV vaccination status did not appear to influence oral HPV prevalence in the sample (*p* = 0.2).

### Female risk factors associated with oral HPV

To determine female risk factors of oral HPV, the behaviours of the female sample were examined in isolation (Table 2). Individuals who were positive for oral HPV smoked more frequently than those who were negative (*p* = 0.05), with post hoc testing revealing a role for cigarettes smoked per day (*p* = 0.1), and increased pack years (*p* = 0.15) in the association. HPV-positive women were also more likely to consume alcohol (*p* = 0.04), but no differences in frequency, unit intake, alcohol type, or binge drinking were observed. Relationship status and self-identified sexual orientation of the female participants did not influence oral HPV status; but open-mouth kissing was significantly associated with female oral prevalence (*p* = 0.029), although no other sexual practice risk factor was associated with oral HPV, including oral sex.

### Male risk factors associated with oral HPV

Similarly, male behaviours were investigated to determine male risk factors of oral HPV (Table 3). Smoking practices (status, frequency, number of cigarettes smoked and/or pack years) were not found to be associated with oral HPV prevalence in the male group. Alcohol consumption practices (status, frequency, unit intake, and/or binge drinking) were also not found to be associated with oral HPV, but mixing multiple types of alcohol was associated with male oral prevalence (*p* = 0.04). In terms of sexual practices, no risk factors were associated with oral HPV, but masturbation was increased amongst HPV-positive individuals. As expected, HPV vaccination could not be examined due to a lack of vaccinated male participants within this group.

### Differences between the HPV-positive male and female samples

To further determine if there were any differences in behaviours influencing oral HPV prevalence between the two genders, the behaviours of the HPV-positive male and female groups were compared (Supplementary Table 2). HPV-positive men were found to drink alcohol more frequently than HPV-positive women (*p* = 0.08) and have a higher unit intake per week (*p* = 0.04). In terms of sexual practices, HPV-positive men were more likely to engage in anal sex (*p* = 0.057) and masturbation (*p* = 0.029) than HPV-positive women. HPV-positive men also engaged in a greater diversity of sexual activity overall than HPV-positive women (*p* = 0.086).

## Discussion

This work set out to describe the prevalence and abundance of oral HPV within a young adult population and identify concordant risk factors. The exploration of the demographics and characteristics of the study population, in conjunction with national statistical databases (Office for National Statistics, NHS Digital, and Natsal-3), suggested that the sample was representative of the wider UK young adult population. Concurrent lifestyle practices observed included smoker status and practices, alcohol consumption, frequency of having sexual intercourse frequency, STD/STI prevalence, numbers of sexual partners, and condom usage [18–22]. We did not capture socioeconomic status (SES), a limitation of this work given the established relationship between SES and both OPSCC incidence and outcomes [23]. We note that the population sampled here are studying for a higher education (HE) degree, which could be suggestive of a higher SES; indeed, this qualification may subsequently lead to a higher occupational status of the participants.

### Prevalence and abundance of oral HPV

The consensus screen described an oral HPV prevalence rate of 22.79% within the sample population, substantially higher than other reported UK rates [14, 15], but similar to other national studies [13, 24, 25]. In support of the published literature [9, 26], high-risk HPV-16 was the most reported viral type within the HPV-positive samples (83.8%); far higher than the second most prevalent viral type, HPV-18 (7.53%), suggesting that HPV-16 is more frequently circulating in the healthy population, as well as dominating within disease. The viral load of HPV-16 was 10–100 fold greater than that of HPV-18; high viral load is a strong predictor of oral HPV persistence [27] and malignant transformation [28]. Surprisingly, we found no difference in oral prevalence or abundance between our self-defined male and female populations despite established reports of higher infections and disease within men [3, 8], suggesting that these gender differences may arise later, and be associated with HPV persistence [12, 27].

### Risk factors for HPV prevalence

The high oral HPV prevalence rate observed here is likely to reflect the age group of the sample population, 92.01% of whom were aged 18–25, and therefore more likely to engage with the behaviours believed to be risk factors for HPV than the general population [18]. Interestingly, HPV-related OPSCC is more common within a higher SES population, whereas HPV-negative OPSCC is more strongly associated with a lower SES [23]; future work exploring the SES of our HE sample will inform the prevalence determinants of HPV infections through to disease in the young adult population.

We had intended to use discriminative models to explore the associations between oral HPV-positivity and viral load with lifestyle behaviours believed to increase the risk of oral HPV infection, but our HPV-positive group (*n* = 93) was too small to permit this. Similarly, post hoc tests were also unable to reveal significant differences between the subgroups when the alpha value was adjusted for multiple comparisons using Bonferroni’s correction, likely due to the sample size. Benjamini–Hochberg ranking instead enabled the identification of important avenues worthy of further investigation. Thus, our data have suggested that associations between HPV-positivity and behavioural risk factors identified in other studies did not always play a role within our sample. For instance, smoking and alcohol use have been linked to oral HPV prevalence [6, 8, 29], but we found no difference in oral prevalence between smokers and non-smokers overall, or no role for alcohol-related behaviours; instead our data indicate that it is the consumption levels that are associated with oral HPV. Indeed, examining our smoking sample herewith reveals that the smoking consumption of the female population influences the association of smoking with HPV. However, few smokers took part in the study, most likely reflecting the significant reduction in young adult smokers over the past 10 years. It is known that smoking does influence oral HPV prevalence and is likely to contribute to persistence and subsequent disease due to damage to the oral epithelium [30]; this work suggests that smoking frequency and pack years, rather than smoker status, should be examined in future studies.

Within the female sample, smoking and alcohol consumption were significant risk factors for oral HPV prevalence. Similarly, mixing of alcohol types, but not other consumption behaviours, was associated with oral HPV prevalence within the male group. In both cases this is complex to explain, but warrants further investigation within the young adult population, and supports the need to examine population subsets to identify nuances in the data between and within genders.

Sexual practices and behaviours have been directly linked to genital HPV prevalence for the past 30 years [18]. However, the sexual practices linked to oral HPV are different to those associated with genital HPV, notably ‘deep-kissing’ and oral sex [29]. In the female sample, open-mouth kissing, particularly, was significantly associated with oral prevalence. Furthermore, we found that more participants within the HPV-positive group engaged in open-mouth kissing and oral sex than within the HPV-negative group. However, we were expecting oral sex to be significantly associated with oral HPV in this sample, given the well-established link between oral sex and oral HPV prevalence [29]. Instead, oral sex was ranked below other sexual behaviours in both the overall HPV-positive sample and also when considering men and women separately. Whilst this could suggest differences in the sexual behaviours of the young adult population, it is more likely that our sample size and statistical approach did not reveal this association – it would be interesting to explore this further in a larger sample..

Conversely, oral HPV prevalence was significantly associated with both masturbation and increasing diversity of sexual acts across the sample population, although we recognise that the association with diverse acts may be driven by that of masturbation. Masturbation was the highest-ranking risk factor for the male population too, although this did not reach significance. Tentative links between masturbation and oral HPV have been made in other studies, referred to as ‘self-inoculation’ [25, 31], but further enquiry is required to determine why masturbation, in particular, correlates with higher oral HPV rates. Within the young adult population, diversity of sexual acts has been associated with increased risk of oral HPV [29], which is supported by these data. Oral HPV-16 was also statistically more prevalent in those who engaged in masturbation and varied sexual activity, suggesting potential transmission of high-risk viral types between genital and oral regions. Indeed, with 28.05% of the HPV-positive group engaging in oral-genital activity, and 74.39% regularly engaging in four or more sexual activities, the transmission of high-risk HPV to multiple anatomical sites is certainly possible [5, 29]. We suggest that, in future, the frequency and diversity of sexual acts should be examined more closely to determine oral-genital HPV transmission routes, alongside longitudinal studies to examine the effects of viral load on oral HPV persistence and clearance.

## Conclusions

This study is one of the first to determine the dominance of oral HPV-16 prevalence, and viral abundance, within young healthy adults. The high HPV prevalence within this sample population suggests that behaviours associated with a young adult population are concordant risk factors for oral HPV, and that studying this sample in isolation, away from the general population, has demonstrated that young adults are a large reservoir for oral HPV. Although we acknowledge that only a small proportion of those with high-risk oral HPV infections will develop OPSCC in later life, young adults should be targeted with public health campaigns highlighting the risks and symptoms of disease onset, such that the morbidity and mortality associated with HPV-positive OPSCC is proactively reduced.

### Supplementary Information

Supplementary Material 1.

Supplementary Material 2.

Supplementary Material 3.

## Data Availability

The datasets used and analysed during the current study are available from the corresponding author on reasonable request.

## Abbreviations

HPV: Human Papillomavirus
NHS: National Health Service
OPSCC: Oropharyngeal Squamous Cell Carcinoma
PCR: Polymerase Chain Reaction
qPCR: Quantitative Polymerase Chain Reaction
STD/STI: Sexually Transmitted Diseases/Infections
