# Supplementary material for: Risk factors associated with oral Human Papillomavirus (HPV) prevalence within a young adult population
Source: BMC Public Health. 2024 Jun 3;24:1485. doi: 10.1186/s12889-024-18977-x (PMC11145846; doi:10.1186/s12889-024-18977-x)
Supplement: Supplementary file 1 — Supplementary Material 1. [file 12889_2024_18977_MOESM1_ESM.docx]

**Supplementary Table 1.** HPV-16 screening results with demographics and lifestyle risk factors.

|  | **HPV-16 +ve**  ***n* = 78** | | **HPV-16 –ve**  ***n* = 330** | | ***p*-value** | **Adjusted *p*-value^c^** |
| --- | --- | --- | --- | --- | --- | --- |
| **Demographics** |  |  |  |  |  |  |
| Aged 18-25 | 73 | (93.59) | 303 | (91.82) | .601 | .2 |
| Aged >25 | 5 | (6.41) | 27 | (8.18) |  |  |
| Ethnicity: White British | 68 | (87.18) | 268 | (81.21) | .214 | .067 |
| Ethnicity: Other White, BAME/BME & Unknown* | 10 | (12.82) | 62 | (18.79) |  |  |
| Gender: Male | 35 | (44.87) | 130 | (39.39) | .375 | .133 |
| Gender: Female | 43 | (55.13) | 200 | (60.61) |  |  |
| Gender: Other | 0 | (0.00) | 0 | (0.00) |  |  |
| **Smoker Status** |  |  |  |  |  |  |
| Current | 14 | (17.95) | 64 | (19.39) | .92 | .2 |
| Former | 6 | (7.69) | 22 | (6.67) |  |  |
| Never | 58 | (74.36) | 244 | (73.94) |  |  |
| **Smoking Frequency** | ***n* = 20** | | ***n* = 81** | |  |  |
| Daily | 13 | (65.00) | 34 | (41.98) | .043b | **.05** |
| 3-5 times/week | 3 | (15.00) | 11 | (13.58) |  |  |
| 1-2 times/week | 2 | (10.00) | 7 | (8.64) |  |  |
| Few times/month | 0 | (0.00) | 16 | (19.75) |  |  |
| Once a month | 0 | (0.00) | 1 | (1.23) |  |  |
| Few times/year | 2 | (10.00) | 12 | (14.81) |  |  |
| **Calculated Smoking Data** | ***n* = 20** | | ***n* = 77** | |  |  |
| No. of cigarettes/day | 7.10 ± 6.01 | (0.01-20.00) | 5.04 ± 5.24 | (0.01-20.00) | .146b | .1 |
| Pack Years | 1.68 ± 2.09 | (0.01-8.00) | 1.87 ± 4.06 | (0.01-27.00) | .232b | .15 |
| **Alcohol Consumption Status** |  |  |  |  |  |  |
| Current | 74 | (94.87) | 287 | (86.97) | .170a | .08 |
| Former | 1 | (1.28) | 12 | (3.64) |  |  |
| Never | 3 | (3.85) | 31 | (9.39) |  |  |
| **Drinking Frequency** | ***n* = 75** | | ***n* = 299** | |  |  |
| Daily | 2 | (2.67) | 7 | (2.34) | .322b | .12 |
| 3-5 times/week | 9 | (12.00) | 28 | (9.36) |  |  |
| 1-2 times/week | 30 | (40.00) | 99 | (33.11) |  |  |
| Few times/month | 13 | (17.33) | 89 | (29.77) |  |  |
| Once a month | 9 | (12.00) | 21 | (7.02) |  |  |
| Few times/year | 12 | (16.00) | 55 | (18.39) |  |  |
| **Types of Alcohol Consumed** | ***n* = 75** | | ***n* = 299** | |  |  |
| ≥2 Types of Alcohol Consumed | 56 | (74.67) | 193 | (64.55) | .097 | .04 |
| **Calculated Data** | ***n* = 63** | | ***n* = 218** | |  |  |
| No. of units/week | 15.93 ± 16.31 | (1.00-71.00) | 14.28 ± 15.68 | (1.00-80.00) | .467b | .16 |
| Binge Drinking | 35 | (55.56) | 115 | (52.75) | .694 | .2 |
| **Relationship Status** |  |  |  |  |  |  |
| Single | 35 | (44.87) | 139 | (42.12) | .861 | .2 |
| Short-term (<1 year) | 13 | (16.67) | 53 | (16.06) |  |  |
| Long-term/Married (≥1 year) | 30 | (38.46) | 138 | (41.82) |  |  |
| **Sexual Orientation** |  |  |  |  |  |  |
| Heterosexual | 66 | (84.62) | 271 | (82.12) | .254a | .029 |
| Homosexual | 5 | (6.41) | 9 | (2.73) |  |  |
| Bisexual | 4 | (5.13) | 31 | (9.39) |  |  |
| Other/Unknown | 3 | (3.85) | 19 | (5.76) |  |  |
| **Sexual Practice Descriptors** |  |  |  |  |  |  |
| Open-Mouth Kissing | 71 | (91.03) | 284 | (86.06) | .241 | .114 |
| Ever had Sexual Intercourse | 68 | (87.18) | 281 | (85.15) | .647 | .114 |
| Within the last year† | 62 | (91.18) | 259 | (92.17) | .787 | .143 |
| STI status† | 7 | (10.29) | 17 | (6.05) | .281 | .057 |
| **Sexual Partners** | ***n* = 68** | | ***n* = 280** | |  |  |
| 1-5 | 46 | (67.65) | 195 | (69.64) | .820a | .171 |
| 6-10 | 11 | (16.18) | 49 | (17.50) |  |  |
| 11-20 | 5 | (7.35) | 19 | (6.79) |  |  |
| >20 | 6 | (8.82) | 17 | (6.07) |  |  |
| **Sexual Activity** | ***n* = 68** | | ***n* = 279** | |  |  |
| Vaginal Sex | 65 | (95.59) | 273 | (97.85) | .386a | .143 |
| Anal Sex | 21 | (30.88) | 74 | (26.52) | .470 | .2 |
| Oral Sex | 64 | (94.12) | 247 | (88.53) | .175 | .086 |
| Foreplay | 62 | (91.18) | 245 | (87.81) | .436 | .171 |
| Masturbation | 57 | (83.82) | 184 | (65.95) | .004 | **.029** |
| **Total Sexual Activities Engaged In** | ***n* = 68** | | ***n* = 279** | |  |  |
| One | 0 | (0.00) | 15 | (5.38) | .044b | **.057** |
| Two | 5 | (7.35) | 18 | (6.45) |  |  |
| Three | 10 | (14.71) | 67 | (24.01) |  |  |
| Four | 36 | (52.94) | 124 | (44.44) |  |  |
| Five | 17 | (25.00) | 55 | (19.71) |  |  |
| **Condom Use** | ***n* = 64** | | ***n* = 264** | |  |  |
| Never | 25 | (39.06) | 80 | (30.30) | .464b | .086 |
| Sometimes (~25%) | 18 | (28.13) | 89 | (33.71) |  |  |
| Mostly (~75%) | 7 | (10.94) | 49 | (18.56) |  |  |
| Always | 14 | (21.88) | 46 | (17.42) |  |  |
| **HPV Vaccination Status** |  |  |  |  |  |  |
| Yes | 30 | (38.46) | 137 | (41.52) | d | |
| No | 40 | (51.28) | 161 | (48.79) |  |  |
| Unsure | 8 | (10.26) | 32 | (9.70) |  |  |

Data shown via count and percentage within HPV status group (%), or mean +/- standard deviation (range). All data analysed using Chi-square tests for categorical observations, unless indicated. Denominators vary across variables because of item non-response. *Grouped for statistical analysis due to large proportion of self-identified White British participants. †Data shown for sexually active group; *n* = 349 (+ve = 68; –ve = 281). ^a^Fisher’s Exact test used due to expected counts being <5. ^b^Mann Whitney U test used for non-parametric continuous or ordinal ranked data. ^c^After Benjamini-Hochberg post-hoc ranking; significance denoted in bold. ^d^Statistical analysis not undertaken. HPV, Human Papillomavirus. STI, sexually transmitted infection.
