# Supplementary material for: Risk factors associated with oral Human Papillomavirus (HPV) prevalence within a young adult population
Source: BMC Public Health. 2024 Jun 3;24:1485. doi: 10.1186/s12889-024-18977-x (PMC11145846; doi:10.1186/s12889-024-18977-x)
Supplement: Supplementary file 2 — Supplementary Material 2. [file 12889_2024_18977_MOESM2_ESM.docx]

**Supplementary Table 2.** HPV positive female and male screening results and lifestyle behaviours.

|  | | **HPV +ve Females**  ***n* = 51** | | | | **HPV +ve Males**  ***n* = 42** | | ***p*-value** | **Adjusted p-value^c^** |
| --- | --- | --- | --- | --- | --- | --- | --- | --- | --- |
| **Smoker Status** | |  | |  | |  |  |  |  |
| Current | | 9 | | (17.65) | | 8 | (19.05) | .871a | .2 |
| Former | | 4 | | (7.84) | | 2 | (4.76) |  |  |
| Never | | 38 | | (74.51) | | 32 | (76.19) |  |  |
| **Smoking Frequency** | | ***n* = 13** | | | | ***n* = 10** | |  |  |
| Daily | | 9 | | (69.23) | | 6 | (60.00) | .496b | .15 |
| 3-5 times/week | | 2 | | (15.38) | | 1 | (10.00) |  |  |
| 1-2 times/week | | 2 | | (15.38) | | 1 | (10.00) |  |  |
| Few times/month | | 0 | | (0.00) | | 0 | (0.00) |  |  |
| Once a month | | 0 | | (0.00) | | 0 | (0.00) |  |  |
| Few times/year | | 0 | | (0.00) | | 2 | (20.00) |  |  |
| **Calculated Smoking Data** | | ***n* = 13** | | | | ***n* = 10** | |  |  |
| No. of cigarettes/day | | 7.95 ± 5.92 | | (0.01-20.00) | | 5.48 ± 5.57 | (0.01-17.00) | .394b | .1 |
| Pack Years* | | 2.98 ± 3.43 | | (0.01-10.00) | | 0.92 ± 0.84 | (0.01-2.00) | .193b | .05 |
| **Alcohol Consumption Status** | |  | |  | |  |  |  |  |
| Current | | 50 | | (98.04) | | 37 | (88.10) | .057a | **.12** |
| Former | | 1 | | (1.96) | | 1 | (2.38) |  |  |
| Never | | 0 | | (0.00) | | 4 | (9.52) |  |  |
| **Drinking Frequency** | | ***n* = 51** | | | | ***n* = 38** | |  |  |
| Daily | | 2 | | (3.92) | | 1 | (2.63) | .014b | **.08** |
| 3-5 times/week | | 1 | | (1.96) | | 8 | (21.05) |  |  |
| 1-2 times/week | | 19 | | (37.25) | | 15 | (39.47) |  |  |
| Few times/month | | 10 | | (19.61) | | 9 | (23.68) |  |  |
| Once a month | | 10 | | (19.61) | | 0 | (0.00) |  |  |
| Few times/year | | 9 | | (17.65) | | 5 | (13.16) |  |  |
| **Types of Alcohol Consumed** | | ***n* = 51** | | | | ***n* = 38** | |  |  |
| ≥2 Types of Alcohol Consumed | | 35 | | (68.63) | | 31 | (81.58) | .167 | .16 |
| **Calculated Data** | | ***n* = 42** | | | | ***n* = 30** | |  |  |
| No. of units/week | | 12.49 ± 14.79 | | (1.00-71.00) | | 20.95 ± 18.56 | (1.00-70.00) | .007b | **.04** |
| Binge Drinking | | 21 | | (50.00) | | 18 | (60.00) | .401 | .2 |
| **Relationship Status** | |  | |  | |  |  |  |  |
| Single | | 21 | | (41.18) | | 20 | (47.62) | .127 | .114 |
| Short-term (<1 year) | | 5 | | (9.80) | | 9 | (21.43) |  |  |
| Long-term/Married (≥1 year) | | 25 | | (49.02) | | 13 | (30.95) |  |  |
| **Sexual Orientation** | |  | |  | |  |  |  |  |
| Heterosexual | | 40 | | (78.43) | | 38 | (90.48) | .391a | .142 |
| Homosexual | | 3 | | (5.88) | | 2 | (4.76) |  |  |
| Bisexual | | 5 | | (9.80) | | 1 | (2.38) |  |  |
| Other/Unknown | | 3 | | (5.88) | | 1 | (2.38) |  |  |
| **Sexual Practice Descriptors** | |  | |  | |  |  |  |  |
| Open-Mouth Kissing | | 49 | | (96.08) | | 37 | (88.10) | .238a | .143 |
| Ever had Sexual Intercourse | | 46 | | (90.20) | | 36 | (85.71) | .536a | .171 |
| Within the last year† | | 45 | | (97.83) | | 31 | (86.11) | .082a | .029 |
| STI status† | | 3 | | (6.52) | | 4 | (11.11) | .694a | .2 |
| **Sexual Partners** | | ***n* = 46** | | | | ***n* = 36** | |  |  |
| 1-5 | | 34 | | (73.91) | | 20 | (55.56) | .107a | .057 |
| 6-10 | | 5 | | (10.87) | | 10 | (27.78) |  |  |
| 11-20 | | 5 | | (10.87) | | 2 | (5.56) |  |  |
| >20 | | 2 | | (4.35) | | 4 | (11.11) |  |  |
| **Sexual Activity** | | ***n* = 46** | | | | ***n* = 36** | |  |  |
| Vaginal Sex | | 46 | | (100.00) | | 33 | (91.67) | .081a | **.114** |
| Anal Sex | | 10 | | (21.74) | | 16 | (44.44) | .028 | **.086** |
| Oral Sex | | 43 | | (93.48) | | 34 | (94.44) | 1a | .171 |
| Foreplay | | 42 | | (91.30) | | 33 | (91.67) | 1a | .2 |
| Masturbation | | 29 | | (63.04) | | 36 | (100.00) | <.001 | **.029** |
| **Total Sexual Activities Engaged In** | | ***n* = 46** | | | | ***n* = 36** | |  |  |
| One | | 1 | | (2.17) | | 0 | (0.00) | .014b | **.057** |
| Two | | 5 | | (10.87) | | 0 | (0.00) |  |  |
| Three | | 10 | | (21.74) | | 5 | (13.89) |  |  |
| Four | | 21 | | (45.65) | | 18 | (50.00) |  |  |
| Five | | 9 | | (19.57) | | 13 | (36.11) |  |  |
| **Condom Use** | | ***n* = 45** | | | | ***n* = 33** | |  |  |
| Never | | 21 | | (46.67) | | 9 | (27.27) | .122b | .086 |
| Sometimes (~25%) | | 12 | | (26.67) | | 10 | (30.30) |  |  |
| Mostly (~75%) | | 4 | | (8.89) | | 8 | (24.24) |  |  |
| Always | | 8 | | (17.78) | | 6 | (18.18) |  |  |
| **HPV Vaccination Status** | |  | |  |  |  |  |  |  |
| Yes | | 34 | | (66.67) | 0 | (0.00) | d | |  |
| No | | 12 | | (23.53) | 38 | (90.48) |  |  |  |
| Unsure | | 5 | | (9.80) | 4 | (9.52) |  |  |  |

Data shown via count and percentage within HPV status group (%), or mean +/- standard deviation (range). All data analysed using Chi-square tests for categorical observations, unless indicated. Denominators vary across variables because of item non-response. *Calculated for smokers that provided information on number of years spent smoking; *n* = 17 (+ve F = 10; +ve M = 7). †Data shown for sexually active group; *n* = 82 (+ve F = 46; +ve M = 36). ^a^Fisher’s Exact test used due to expected counts of <5. ^b^Mann Whitney U test used for non-parametric continuous or ordinal ranked data. ^c^After Benjamini-Hochberg post-hoc ranking; significance denoted in bold. ^d^Statistical analysis not possible. HPV, Human Papillomavirus. STI, sexually transmitted infection.
