# Supplementary material for: Risk factors associated with oral Human Papillomavirus (HPV) prevalence within a young adult population
Source: BMC Public Health. 2024 Jun 3;24:1485. doi: 10.1186/s12889-024-18977-x (PMC11145846; doi:10.1186/s12889-024-18977-x)
Supplement: Supplementary file 3 — Supplementary Material 3. [file 12889_2024_18977_MOESM3_ESM.docx]

**Questionnaire For**: **A** **Research Study Investigating the Prevalence of Infection of the Human Papilloma Virus (HPV) and Merkel Cell Polyomavirus (MCPyV) in the Adult Population**

This questionnaire is forming part of an investigation into determining the levels of HPV and MCPyV infection in adults. It will help the research team understand if particular lifestyle choices that people undertake can increase a person’s risk of contracting one or both of these viral infections.

The questionnaire is confidential and anonymous, and your unique identification number is only to ensure that each donated sample and questionnaire can be linked together. Some of the questions are of a personal nature; please try to answer these questions in a truthful and open manner.

**Question 1: What is your age?** 18-25 26-35 36-45 46-55 56-65 66-75 >75

**Question 2: Are you male or female?** Male Female Other

**Question 3: What is your current relationship status?** *(Tick* **ALL** *that apply)*

Married and/or in long-term relationship (>1 yr)

In a short-term relationship (<1yr)

Single

Divorced and/or Widowed

**Question 4: What is your ethnic background & nationality?** .................................. (E.g. White British)

**Question 5: Do you have any existing diagnosed health condition(s) (incl. allergies)?**

YES NO *(If* ***NO****, please go to Question 6)*

- ***Where on your body does it affect?***  *(Tick* **ALL** *that apply)*

Integumentary (I.e. Skin, Hair & Nails) Digestive/Excretory

Respiratory Muscular & Skeletal

Reproductive (Internal & External) Nervous

Circulatory (Incl. Blood) Endocrine (I.e. Hormones)

Lymphatic & Immunological Renal/Urinary

Mental/Psychological Other/Don’t Know

- ***What is this existing diagnosed health condition(s) and the year(s) of medical diagnosis?*** *(If you do not wish to disclose the information, please state below)*

**Condition(s):** *(E.g. Asthma) ……………………………………………………………………….……………………………………………………………………….……………………………………………………………………….……………………………………………………………………….*

**Exact Diagnosis Year(s):** (*E.g. 2004*) *……………………………………………………………………….* *……………………………………………………………………….* *……………………………………………………………………….* *……………………………………………………………………….*

**QUESTIONS 6-7 ARE FOR CURRENT SMOKERS (INCL. SOCIAL SMOKERS)**  **If you have NEVER smoked anything, go to Question 10**

**Question 6: How often do you smoke cigarettes & how many?**  *(Tick* **ONE** *that applies)*

Daily

3-5 times/week

1-2 times/week

Few times a month

Once a month

Infrequently (few times a year)

How many: …………………..

How many: …………………..

How many: …………………..

How many: …………………..

How many: …………………..

How many: …………………..

- ***For how many years have you smoked?*** *Approx. ……………..…..Years*

**Question 7: Do you smoke anything else other than cigarettes?** *(If* **NO***, disregard Question 7)* *(E.g. cigars, pipes, cannabis, shisha and/or other)*

- ***Please state what****: ……………………………………………………………………………….*
- ***How often do you smoke these items & how many?*** *(Tick* **ONE** *that applies)*

Daily

3-5 times/week

1-2 times/week

Few times a month

Once a month

Infrequently (few times a year)

How many: …………………..

How many: …………………..

How many: …………………..

How many: …………………..

How many: …………………..

How many: …………………..

- ***For how many years have you smoked these items?*** *Approx. ……………..…..Years*

**QUESTIONS 8-9 ARE FOR PREVIOUS SMOKERS**

**Question 8: How often did you smoke cigarettes & how many?**  *(Tick* **ONE** *that applies)*

Daily

3-5 times/week

1-2 times/week

Few times a month

Once a month

Infrequently (few times a year)

How many: …………………..

How many: …………………..

How many: …………………..

How many: …………………..

How many: …………………..

How many: …………………..

- ***For how many years did you smoke?*** *Approx. ……………..…..Years*

**Question 9: Did you smoke anything else other than cigarettes?** *(If* **NO***, disregard Question 9)* *(E.g. cigars, pipes, cannabis, shisha and/or other)*

- ***Please state what****: ……………………………………………………………………………….*
- ***How often did you smoke these items & how many?*** *(Tick* **ONE** *that applies)*

Daily

3-5 times/week

1-2 times/week

Few times a month

Once a month

Infrequently (few times a year)

How many: …………………..

How many: …………………..

How many: …………………..

How many: …………………..

How many: …………………..

How many: …………………..

- ***For how many years did you smoke these items?*** *Approx. ……………..…..Years*

**QUESTION 10-12 RELATE TO ALCOHOL CONSUMPTION If you have NEVER drank alcohol, go to Question 13**

**Question 10: How often do you currently drink alcohol & how much?** *(Tick* **ONE** *that applies) (E.g. A bottle of 9% red wine & 2 pints of cider)*

Daily

3-5 times/week

1-2 times/week

Few times a month

Once a month

Few times a year OR Don’t currently

How much: ……………………………………………………

How much: ……………………………………………………

How much: ……………………………………………………

How much: ……………………………………………………

How much: ……………………………………………………

How much: …………………..………………………………

- ***What type(s) of alcohol do you currently drink the most?*** *(Tick* **ALL** *that apply)*

N/A Beer/Ale Wine Lager Neat Spirits Spirits & Mixer(s)

Cider Alcopops (E.g. WKD) Other -*Please state what*: ……………………………………….

**Question 11: If you currently drink, did you used to drink more heavily*?***

YES NO *(If* ***NO****, go to Question 13)* N/A

- ***If so, how often did you drink alcohol & how much?*** *(Tick* **ONE** *that applies) (E.g. A bottle of 9% red wine & 2 pints of cider)*

Daily

3-5 times/week

1-2 times/week

Few times a month

Once a month

Few times a year

How much: ……………………………………………………

How much: ……………………………………………………

How much: ……………………………………………………

How much: ……………………………………………………

How much: ……………………………………………………

How much: …………………..………………………………

- ***What type(s) of alcohol did you drink the most?*** *(Tick* **ALL** *that apply)*

Beer/Ale Wine Lager Neat Spirits Spirits & Mixer(s)

Cider Alcopops (E.g. WKD) Other -*Please state what*: ……………………………………….

**Question 12: If you do not currently drink but you used to, how often did you & how much?** *(E.g. A bottle of 9% red wine & 2 pints of cider)*  *(Tick* **ONE** *that applies)*

Daily

3-5 times/week

1-2 times/week

Few times a month

Once a month

Few times a year

How much: ……………………………………………………

How much: ……………………………………………………

How much: ……………………………………………………

How much: ……………………………………………………

How much: ……………………………………………………

How much: …………………..………………………………

- ***What type(s) of alcohol did you drink the most?*** *(Tick* **ALL** *that apply)*

Beer/Ale Wine Lager Neat Spirits Spirits & Mixer(s)

Cider Alcopops (E.g. WKD) Other -*Please state what*: ……………………………………….

**Question 13: Do you engage in open-mouth kissing** (with and without tongue)**?**

YES NO

**Question 14: Are you? Heterosexual** *(Tick* **ONE** *that applies)*

**Homosexual**

**Bisexual**

**Other** *Please give details:* ...........................................................

*The following questions are of a personal nature, but are essential for the successful completion of the aims of this investigation and as such, please answer each question truthfully. You may wish to ensure you answer these questions in an area that cannot be observed for your own privacy.*

**QUESTION 15-21 RELATE TO SEXUAL PRACTICE If you have NEVER had sexual intercourse, go to Question 22**

**Question 15: How many sexual partners have you had?** *(Tick* **ONE** *that applies)*

1-5 6-10 11-20 21-30 31-40 41-50 50+

**Question 16: Are you in a monogamous relationship?**

YES -*If so, how long for?* ...........Years.......... Months NO

**Question 17: Have you had sexual intercourse within the last year?** YES NO

**Question 18: Which sexual activities have you performed/currently perform?** *(Tick* **ALL** *that apply)*

Vaginal sex Anal sex Oral sex Foreplay Masturbation

**Question 19: On a scale of 1 to 5 (1 = Always & 5 = Never), how often in the last year, have you used a condom?** *(Tick* **ONE** *that applies)*

1 2 3 4 5 *-If Never, why? …………………………………….*

**Question 20: On a scale of 1 to 5 (1 = None & 5 = All), how many of your total number of sexual partners were one night stands?** *(Tick* **ONE** *that applies)*

1 2 3 4 5

**Question 21: Have you ever tested positive for a sexual transmitted disease (STD)?**

YES -*If so, what type?* ................................................................ NO

**Question 22: Have you ever been diagnosed with a HPV infection?**

YES *-If so, when?* ................................................................ NO

**Question 23: Have you been diagnosed with a HPV-related cancer and/or cancer precursor?**

YES *-If so, what type?* (E.g. CIN Stage II) .................................................................. NO

**Question 24: Have you had the HPV vaccination?** YES NO Unsure

- ***Did you receive it via:*** National Health Service (NHS)

Private UK health-care

Private overseas health-care

Public overseas health-care

**Question 25: Do you use/have you ever used sunbeds for tanning?**  YES NO

- ***If YES, how often do/did you use sunbeds for tanning?*** *(Tick* **ONE** *that applies)*

Every week

Every fortnight

Once a month

Once every 3 months

Once every 6 months

Infrequently (few times a year)

**Question 26: Is there a history of cancer in your family?** YES NO Unsure

- ***If YES, please state what type(s):*** ………………………………………………………………………………………….

**-End of Questionnaire-**

**Thank You for Your Participation**
